# Effects of Straw Management and Nitrogen Application Rate on Soil’s Physicochemical Properties and Rice Yield in Saline–Sodic Paddy Fields

**DOI:** 10.3390/plants13243475

**Published:** 2024-12-11

**Authors:** Cheng Ran, Jiaquan Li, Ya Gao, Yaoru Xie, Yangyang Li, Jiguo Yang, Yanqiu Geng, Liying Guo, Dapeng Gao, Xiwen Shao

**Affiliations:** 1 Heyuan Branch, Guangdong Laboratory for Lingnan Modern Agriculture, Heyuan 517000, China; jlndrc@126.com (C.R.); dtsys_lijiaquan@163.com (J.L.); gy_elegance@163.com (Y.G.); yaoruxie@iclould.com (Y.X.); 17835683214@163.com (Y.L.); detatougao@163.com (J.Y.); 2 Agronomy College, Jinlin Agricultural University, Changchun 130118, China; wangyibeiyongyx@163.com (Y.G.); tougaobeiyongyx@163.com (L.G.); jlndsxw@126.com (X.S.); 3 College of Life Sciences, Linyi University, Linyi 276000, China

**Keywords:** rice, straw return, saline–sodic soil, soil aggregates, soil salinity

## Abstract

Straw return plays a vital role in crop yield and sustainable agriculture. Extensive research has focused on the potential to enhance soil fertility and crop yield through straw return. However, the potential impacts of straw return on saline–sodic soils have been relatively neglected due to the unfavorable characteristics of saline–sodic soils, such as high salinity, poor structure, and low nutrient contents, which are not conducive to crop growth. Therefore, a two-year field experiment was conducted to assess the effects of straw management (retention or removal) with nitrogen fertilizers (0, 90, 180, 270, and 360 kg N ha^−1^) on soil aggregates, soil chemical properties, and rice yields in saline–sodic soil. The results showed that straw return with nitrogen fertilization significantly decreased the soil exchange sodium percentage (ESP) and the percentage and organic carbon contribution of silty clay particles and also significantly increased the soil aggregate stability, organic matter (SOM), and percentage and organic carbon contribution of macroaggregates. However, there was no significant difference between 270 kg N ha^−1^ and 360 kg N ha^−1^ for all soil indicators under straw return. Straw return significantly increased rice grain yield by 5.77% (two-year average) compared to straw removal. The highest grain yield of 8.01 t ha^−1^ (two-year average) was obtained from straw return combined with 270 kg N ha^−1^. However, since this experiment was conducted for only two years, the positive effects of long-term straw return on soil and rice yield could have been greater. Therefore, the application of 270 kg N ha^−1^ in the early stages of straw return is a promising management practice for improving saline–sodic soils and increasing rice yields.

## 1. Introduction

Soil salinization is considered the second most important type of soil degradation after soil erosion [1], causing an economic loss of at least USD 27 billion per year to global agricultural production [2]. The total area of land affected by salinization globally is about 1 billion hectares, and there has been no discernible increasing trend in the last few decades [3]. China is one of the countries more seriously affected by salinization, with a total saline–alkaline land area of about 1.0 × 10^9^ ha, accounting for 10 per cent of the world’s saline–alkaline land area [4]. The Songnen Plain in northeastern China is one of the world’s three largest saline–sodic soil distribution areas, with an estimated saline–sodic land area of more than 3.73 million hectares [5]. The local matrices, topographic features, climatic conditions, and anthropogenic factors contribute to the formation and evolution of soil salinization [6]. Saline–sodic soils contain large amounts of soluble salts, NaHCO_3_ and Na_2_CO_3_ being the main salts, and soil pH is mostly above 8.5 [7]. Due to their poor physicochemical properties and low nutrient content, saline–sodic soils are unsuitable for most crops [8]. In recent decades, with the rapid development of the global economy, the area of arable land has gradually decreased [9]. At the same time, with a world population of up to 8 billion, ensuring stable food production with limited arable land will be a huge challenge. In recent years, the frequent occurrence of extreme weather caused by climate change has led to a sharp rise in global food prices and triggered food crises in some countries [10]. As an important reserve land resource for food production, saline–alkaline land should play an important role in ensuring national food security in the context of the global food crisis.

Rice (*Oryza sativa* L.) is the staple food for more than half of the world’s population and the most important grain crop for Asian people. Planting rice keeps soil flooded for long periods of time, which not only accelerates salt leaching from soil but also increases the abundance of microorganisms and promotes the accumulation of nutrients and thus an improvement in salt–alkaline soil [11]. Rice has become one of the major crops in saline–sodic areas due to the better amelioration effect of growing rice on saline–sodic soils [12]. However, due to the problems of poor soil physical properties, a high degree of salinization, and low nutrient content in the region, the growth and development of rice are seriously affected, resulting in low rice yield and very low economic efficiency, which seriously restricts the development of local agriculture [13]. Improving saline–sodic soils and reducing the damage of saline stress on rice are key to the rational development and utilization of such soils.

Crop straw is an abundant, renewable, and inexpensive resource, rich in organic carbon and various nutrients required by crops [14]. It is well known that straw recycling can improve the productivity and sustainability of agro-ecosystems by improving soil structure and increasing the availability of organic matter and nutrients [15,16]. However, the effects of straw on crop yields are slow and variable [17]. Straw tends to increase the incidence of pests and diseases [18]. Straw consumes large amounts of available nitrogen in soil during degradation, leading to crop nitrogen deficiency and lower crop yields, especially in infertile soils [19]. As a result, farmers in saline–sodic areas are generally reluctant to incorporate straw return into their farming systems. Singh and Sharma [20] suggested that more nitrogen fertilizer may need to be applied under straw return compared to the recommended nitrogen levels for cropland with straw removed. Although mineral nitrogen may be temporarily fixed by microorganisms when straw is added as an available carbon source, the nitrogen produced by straw may make an important contribution to the subsequent crop nitrogen uptake and soil nitrogen fixation [21]. In addition, nitrogen fixation using straw return can reduce nitrogen loss from soil and delay the release of nitrogen to the later stages of crop growth, thus improving the relationship between soil nitrogen supply and plant nitrogen demand [22,23]. Therefore, to improve saline–sodic soils using straw return, it is necessary to understand the effect of the combined application of straw return and nitrogen fertilizer on the physicochemical properties of this type of soil.

Our hypothesis is that straw return combined with nitrogen fertilizer application can effectively improve saline–sodic paddy soils and enhance the productivity of saline–sodic paddy fields, and the resulting combination of straw return and suitable nitrogen fertilizer has greater potential. In order to check this hypothesis, the effects of straw return and nitrogen fertilizer combination on saline–sodic soil and rice yield were investigated in a two-year consecutive field orientation experiment in saline–sodic rice fields. The objectives of this study were as follows: (1) to clarify the effects of straw return and nitrogen fertilizer application on the bulk density, aggregates, electrical conductivity, pH, cation exchange capacity, exchangeable sodium percentage, exchangeable sodium, soil salinity, soil nutrients, and rice yield of saline–sodic soils; (2) to clarify the effects of straw return and nitrogen fertilizer application on the distribution and percentage of SOC in the aggregates of saline–sodic soils; and (3) to determine whether rice straw can be used as an organic soil amendment to improve saline–sodic soils to increase rice yield and to explore the optimum amount of nitrogen fertilizer to be applied to straw return to improve saline–sodic soils.

## 2. Results

### 2.1. Soil Bulk Density and Total Porosity

**Figure 1:**
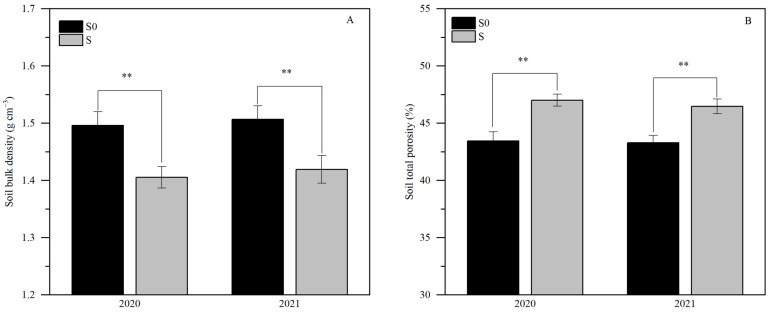
The effect of straw return on soil bulk density (**A**) and total porosity (**B**). S0, S, and ** denote straw removal, straw retention, and *p* < 0.01, respectively.

The analysis of variance (ANOVA) showed that the soil bulk density and total porosity were only affected by the significant (*p* < 0.01) effect of straw return (Figure S1). The soil bulk density and total porosity were 1.50 g cm^−3^ and 43.36% (two-year average) in the straw removal treatment, respectively (Figure 1). In the straw return treatment, the soil bulk density and total porosity were 1.41 g cm^−3^ and 46.69% (averaged over two years), respectively (Figure 1). Compared with straw removal, straw return significantly decreased the soil bulk density by 5.94% (two-year average); meanwhile, straw return also significantly increased the soil total porosity by 7.78% (two-year average).

### 2.2. Soil Aggregate Size Distribution

**Figure 2:**
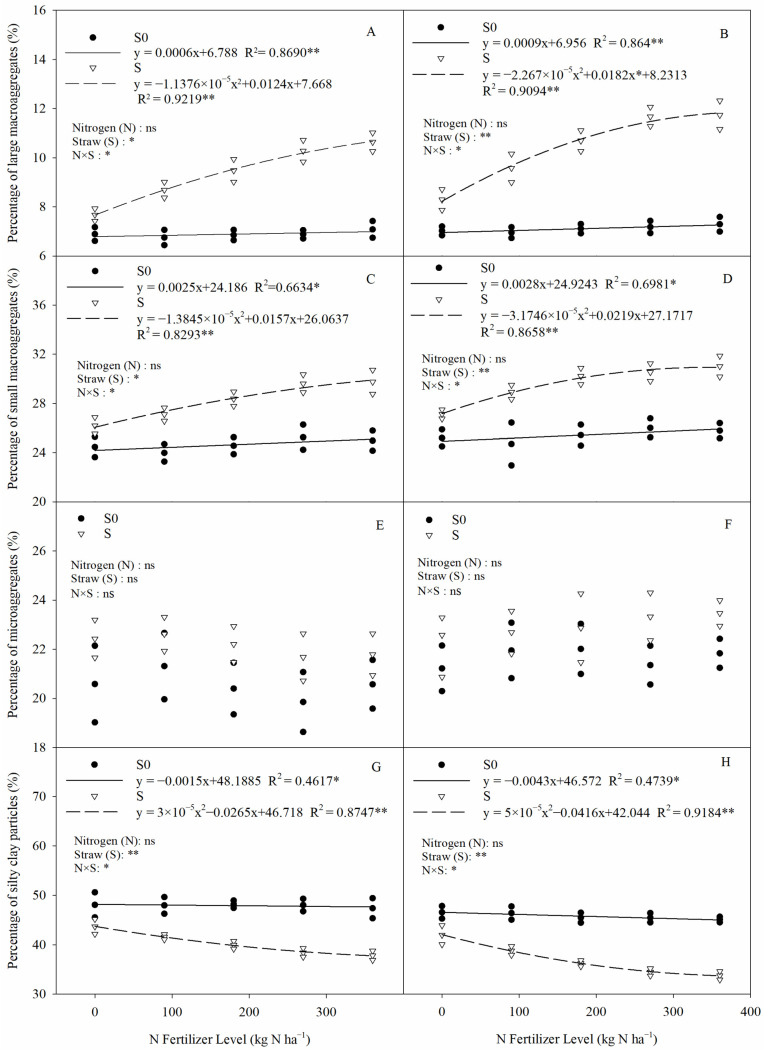
The relationship between nitrogen fertilizer application rate and soil aggregates for (**A**,**C**,**E**,**G**) 2020 and (**B**,**D**,**F**,**H**) 2021. Two-way ANOVA was used to investigate the effects of straw, nitrogen, and their interactions (N × S). S0, S, *, **, and ns denote straw removal, straw retention, *p* < 0.05, *p* < 0.01, and *p* > 0.05, respectively.

Soil aggregates in saline–sodic paddy soils were mainly dominated by aggregates with a particle size of <0.053 mm, accounting for 33.77–48.07%, while >2 mm aggregates accounted for the smallest proportion, at only 6.89–11.74% (Figure 2). The percentage of large macroaggregates, small macroaggregates, and silty clay particles in saline–sodic rice field soils was significantly affected by straw return and straw × nitrogen fertilizer interactions (Figure 2). Compared with straw removal, straw return significantly increased the percentage of large and small macroaggregates by 41.00% and 15.39%, respectively (two-year average); at the same time, straw return significantly decreased the percentage of silty clay particles by 17.52% (two-year average). In straw return, nitrogen fertilizer showed a significant quadratic relationship with macroaggregates and silty clay particles, but soil aggregates were not significantly different between 270 kg N ha^−1^ and 360 kg N ha^−1^. In addition, the application of nitrogen fertilizer alone had no significant influence on the aggregates. This suggests that straw return favors the formation of larger soil aggregates, whereas nitrogen application may favor straw degradation but does not affect the size of aggregates.

### 2.3. Soil Aggregate Stability

**Figure 3:**
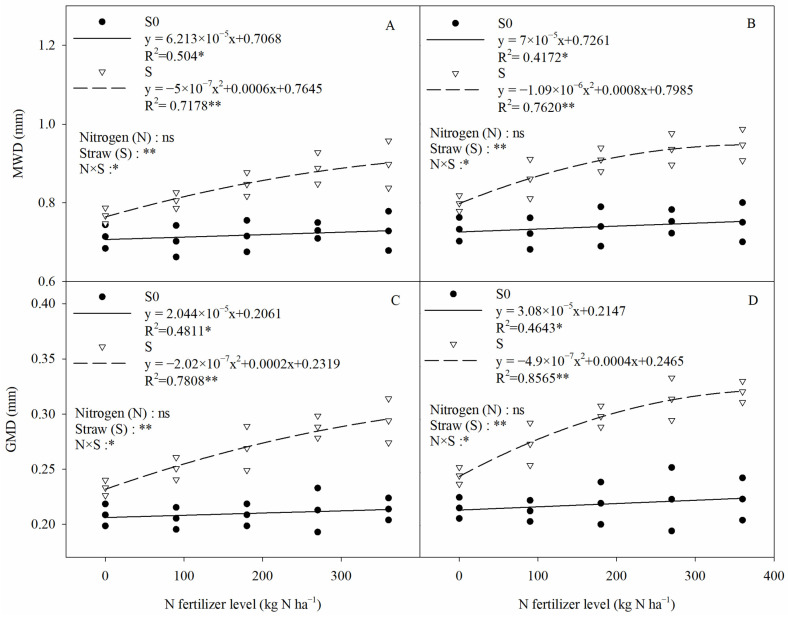
The relationship between soil aggregate stability and nitrogen application rates. MWD and GMD denote the mean weight diameter and geometric mean diameter of the soil, respectively. (**A**,**B**) MWD, (**C**,**D**) GMD, (**A**,**C**) 2020, (**B**,**D**) 2021. Two-way ANOVA was used to investigate the effects of straw, nitrogen, and their interactions (N × S). S0, S, *, **, and ns denote straw removal, straw retention, *p* < 0.05, *p* < 0.01, and *p* > 0.05, respectively.

The mean weight diameter (MWD) and geometric mean diameter (GMD) are often used to evaluate soil aggregate stability. Straw (S) and straw with nitrogen fertilizer (N × S) had significant effects on the MWD and GMD of saline–sodic paddy soils (Figure 3). Straw return significantly increased aggregate MWD and GMD by 22.26% and 27.12%, respectively, compared with straw removal. In straw return, there was a significant negative quadratic relationship between soil aggregate stability and nitrogen fertilizer, and there was no significant difference in soil aggregate GMD and MWD between 270 kg N ha^−1^ and 360 kg N ha^−1^.

### 2.4. Soil Chemical Properties

**Figure 4:**
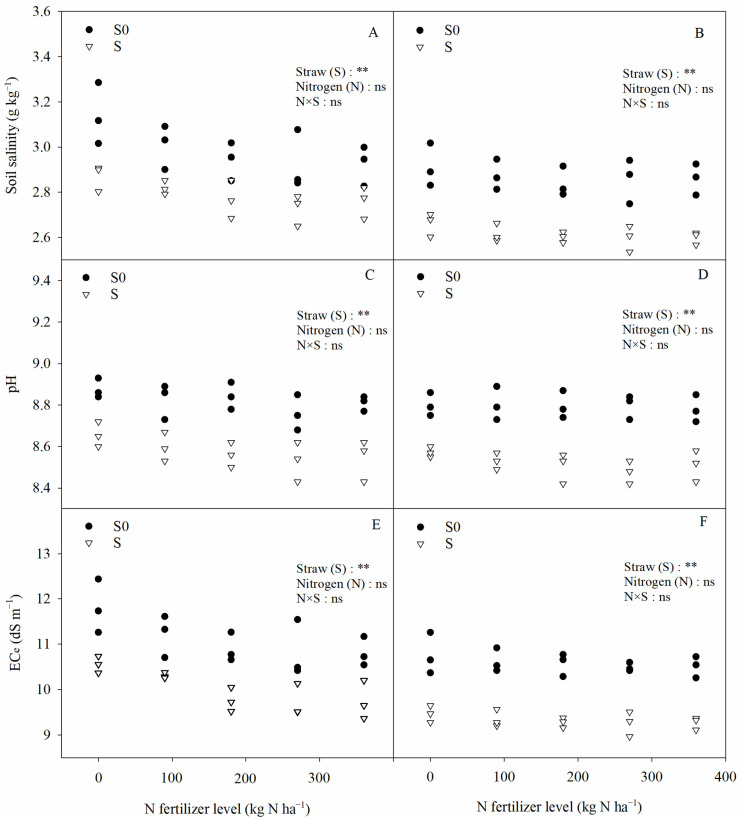
The relationships between soil salinity, pH, electrical conductivity of the soil saturation extract (EC_e_), and nitrogen application rates. (**A**,**C**,**E**) 2020, (**B**,**D**,**F**) 2021. Two-way ANOVA was used to investigate the effects of straw, nitrogen, and their interactions (N × S). S0, S, **, and ns denote straw removal, straw retention, *p* < 0.01, and *p* > 0.05, respectively.

**Figure 5:**
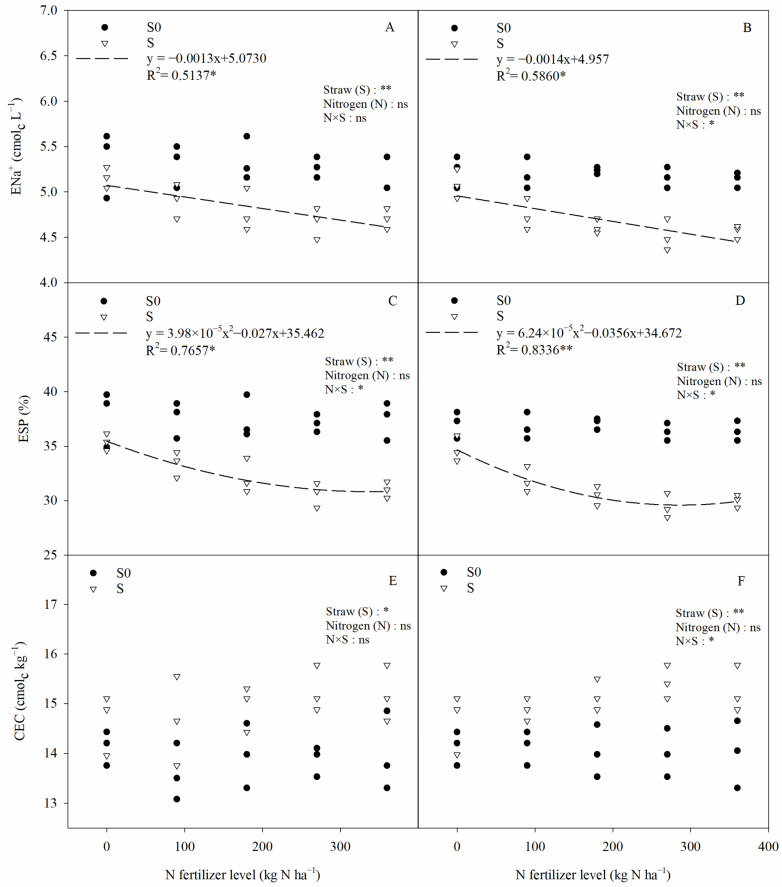
The relationships between the soil ENa^+^, ESP, CEC and nitrogen application rates. CEC, ENa^+^, and ESP represent the soil cation exchange capacity, exchangeable sodium and exchangeable sodium percentage, respectively. (**A**,**C**,**E**) 2020, (**B**,**D**,**F**) 2021. Two-way ANOVA was used to investigate the effects of straw, nitrogen, and their interactions (N × S). S0, S, *, **, and ns denote straw removal, straw retention, *p* < 0.05, *p* < 0.01, and *p* > 0.05, respectively.

Straw return significantly affected the soil salinity, pH, electrical conductivity of the soil saturation extract (EC_e_), exchangeable sodium (ENa^+^), cation exchange capacity (CEC), and exchangeable sodium percentage (ESP) in saline–sodic paddy fields, whereas nitrogen fertilizer had no significant effect on any of the above indicators (Figure 4 and Figure 5). Compared with straw removal, straw return significantly reduced the soil salinity, pH value, EC_e_, ENa^+^, and ESP by 7.73%, 2.98%, 10.92%, 8.96%, and 13.79%, respectively (two-year average); at the same time, it also significantly increased the soil CEC by 5.71% (two-year average). Although straw return had a significant effect on soil chemical properties, N × S only had a significant effect on ESP. Nitrogen fertilizer showed a significant quadratic relationship with ESP and a negative relationship with ENa^+^ under straw return conditions (Figure 5).

### 2.5. Soil Nutrients

**Figure 6:**
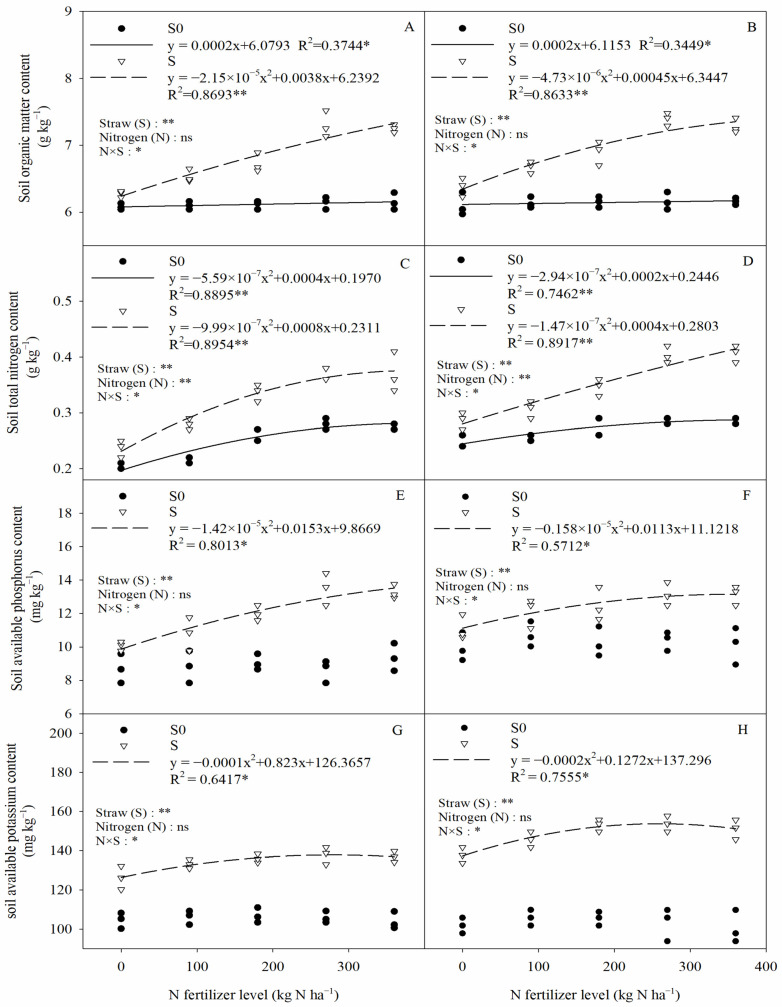
The relationship between soil nutrients and nitrogen fertilization. (**A**,**C**,**E**,**G**) 2020, (**B**,**D**,**F**,**H**) 2021. Two-way ANOVA was used to investigate the effects of straw, nitrogen, and their interactions (N × S). S0, S, *, **, and ns denote straw removal, straw retention, *p* < 0.05, *p* < 0.01, and *p* > 0.05, respectively.

The soil organic matter (SOM), total nitrogen (TN), available phosphorus (AP), and available potassium (AK) in the saline–sodic paddy field were significantly influenced by straw (S), as well as by straw and nitrogen fertilizer interactions (N × S) (Figure 6). In straw removal treatments, significant separation of soil nutrients occurred after straw return, which indicated that there were significant changes in soil nutrients after straw return. Compared to straw removal treatment, straw return significantly increased SOM, TN, AP, and AK by 11.98%, 28.48%, 22.38%, and 35.75%, respectively (two-year average). In addition, the application of nitrogen fertilizer alone only significantly influenced the soil TN content, while it had no significant influence on SOM, AP, or AK. In the straw return treatment, there was a significant negative quadratic relationship between soil nutrients and nitrogen fertilization, and no significant difference in soil nutrients between 270 kg N ha^−1^ and 360 kg N ha^−1^. This suggests that the application of nitrogen fertilizer under the condition of straw return can improve soil nutrients, but there may be a threshold value beyond which nitrogen fertilizer application is counterproductive.

### 2.6. SOC Contents in Aggregates with Different Particle Sizes

**Figure 7:**
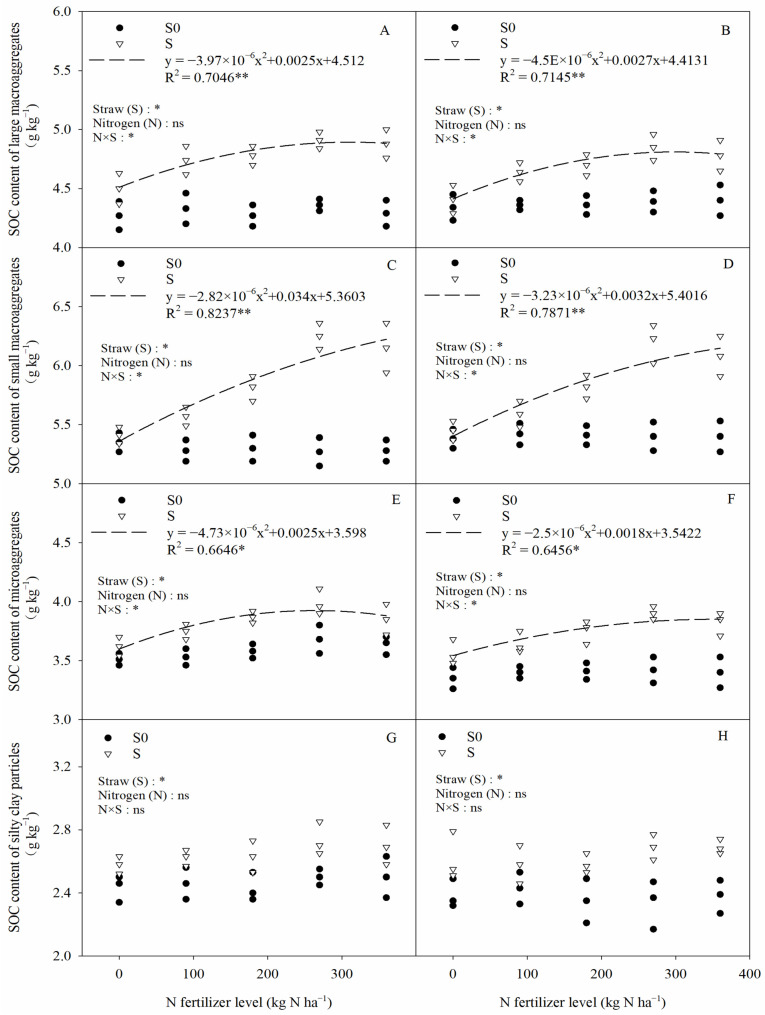
The relationship between the soil organic carbon (SOC) content of soil aggregates with different particle sizes and nitrogen fertilization conditions. (**A**,**C**,**E**,**G**) 2020, (**B**,**D**,**F**,**H**) 2021. Two-way ANOVA was used to investigate the effects of straw, nitrogen, and their interactions (N × S). S0, S, *, **, and ns denote straw removal, straw retention, *p* < 0.05, *p* < 0.01, and *p* > 0.05, respectively.

The SOC contents in different-particle-size aggregates in saline–sodic paddy soil after straw return are shown in Figure 7. Among all the aggregates, small macroaggregates (0.25–2 mm) had the highest SOC contents, followed by large macroaggregates (>2 mm) and then microaggregates (0.25–0.053 mm), while silty clay particles (<0.053 mm) had the lowest SOC content. Straw return significantly increased the SOC content of large macroaggregates, small macroaggregates, microaggregates, and silty clay particles by 8.82%, 8.55%, 6.13%, and 3.35%, respectively (two-year average) compared to straw removal. This suggests that straw return mainly increased the SOC content in macroaggregates (>0.25 mm). Under the straw return condition, the SOC contents of large macroaggregates, small macroaggregates, microaggregates, and silty clay particles increased by 7.41%, 8.66%, 5.68%, and 5.17%, respectively (two-year average) in the nitrogen application treatments compared with 0 kg N ha^−1^. In addition, the interaction (N × S) between straw return and nitrogen fertilization significantly influenced the SOC content of >0.053 mm aggregates, whereas nitrogen fertilization did not have a significant influence on the SOC content of aggregates at any particle size. In straw return, the SOC content of >0.053 mm aggregates showed a significant negative quadratic relationship with nitrogen fertilizer application, and there was no significant difference between nitrogen application of 270 kg N ha^−1^ and 360 kg N ha^−1^. This indicates that excessive nitrogen fertilizer application in straw return does not promote SOC accumulation in aggregates.

### 2.7. Contribution of Soil Aggregate Organic Carbon

**Figure 8:**
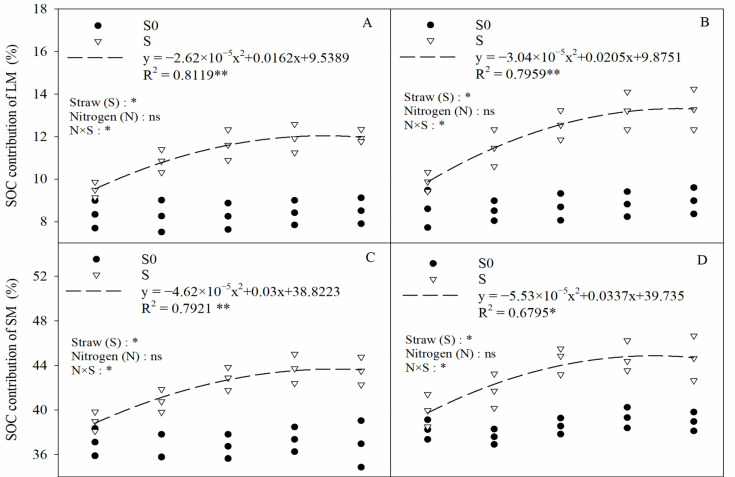

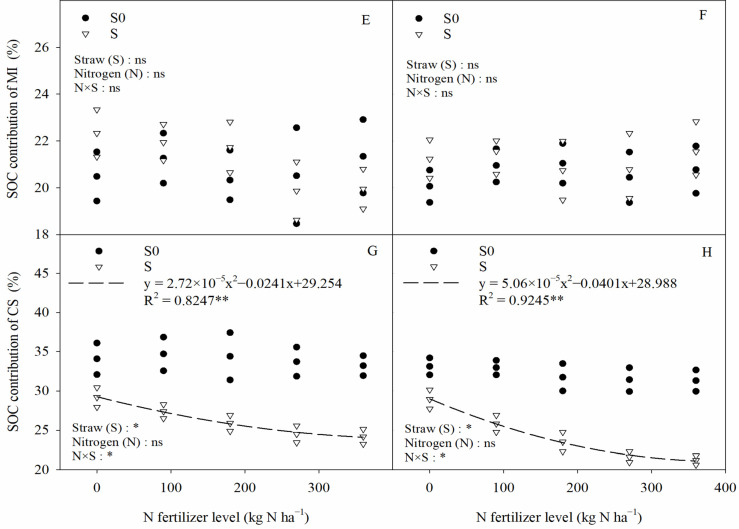
The relationship between the organic carbon (SOC) contribution of soil aggregates of different particle sizes and conditions of nitrogen fertilization. (**A**,**C**,**E**,**G**) 2020, (**B**,**D**,**F**,**H**) 2021. Two-way ANOVA was used to investigate the effects of straw, nitrogen, and their interactions (N × S). LM, SM, MI, and CS represent >2 mm aggregates, 0.25–2 mm aggregates, 0.053–0.25 mm aggregates, and <0.053 mm aggregates, respectively. S0, S, *, **, and ns denote straw removal, straw retention, *p* < 0.05, *p* < 0.01, and *p* > 0.05, respectively.

As a whole, the SOC contribution of small macroaggregates was the highest—as high as 35.75–44.66%—followed by silt clay particles with contributions ranging from 21.19 to 34.08%, while the SOC contribution of large macroaggregates was the lowest, ranging from 8.25 to 13.29% (Figure 8). Straw and the interaction of straw and nitrogen fertilizer (N × S) significantly influenced the SOC contribution of macroaggregates and silty clay particles. Compared with straw removal, straw return treatment significantly increased the SOC contribution of large macroaggregates and small macroaggregates by 36.62% and 11.71%, respectively (two-year average) and also significantly reduced the SOC contribution of silty clay particles by 23.72% (two-year average). The application of nitrogen fertilizer only had no significant influence on the SOC contribution of aggregates of different particle sizes. In straw return, the SOC contribution of macroaggregates and silty clay particles showed a significant quadratic relationship with nitrogen fertilizer; meanwhile, there was no significant difference in SOC contribution between 270 kg N ha^−1^ and 360 kg N ha^−1^. This indicated that the excessive application of nitrogen fertilizer did not increase the organic carbon contribution of aggregates under straw return conditions.

### 2.8. Rice Yield

**Figure 9:**
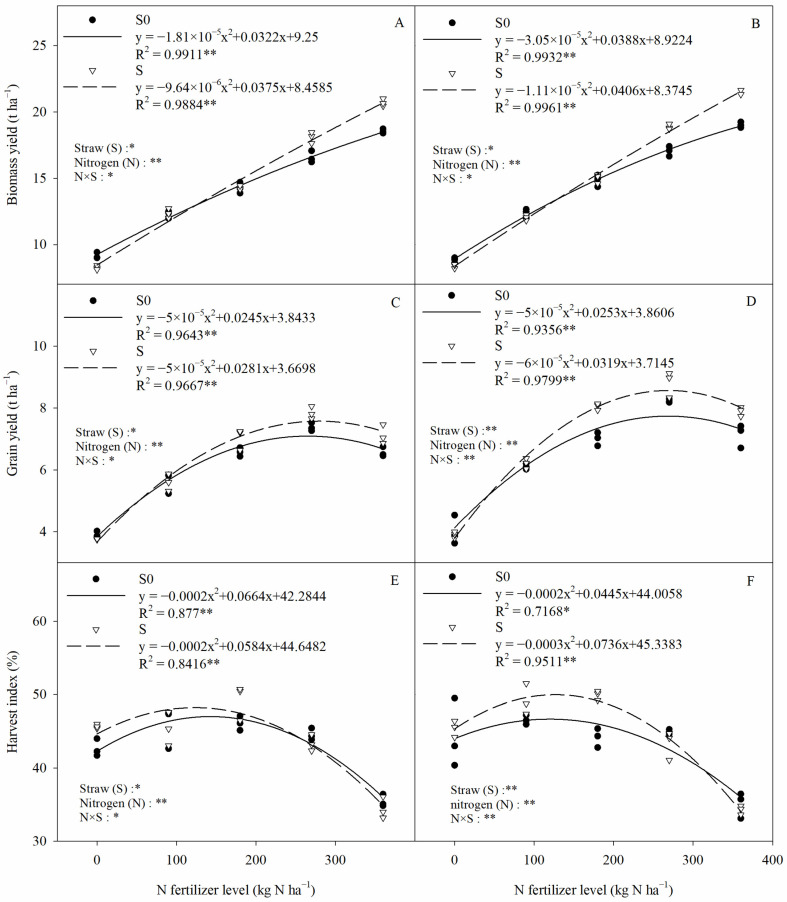
The relationship between rice biomass yield, grain yield, harvest index, and nitrogen fertilization. (**A**,**C**,**E**) 2020, (**B**,**D**,**F**) 2021. Two-way ANOVA was used to investigate the effects of straw, nitrogen, and their interactions (N × S). S0, S, *, and ** denote straw removal, straw retention, *p* < 0.05, and *p* < 0.01, respectively.

Nitrogen fertilizer (N), straw (S), and their interaction (N × S) had a significant influence on the rice biomass yield, grain yield, and harvest index (Figure 9). There was a significant negative quadratic relationship between the rice biomass yield, grain yield, harvest index, and nitrogen fertilizer. The highest grain yield of rice was measured at 270 kg N ha^−1^ with and without straw return, at 7.52 t ha^−1^ (S0) and 8.01 t ha^−1^ (S), respectively. The grain yield and harvest index were significantly lower at 360 kg N ha^−1^ than at 270 kg N ha^−1^ under the same straw regime. This suggests that the excessive application of nitrogen fertilizer can negatively affect rice yield. Compared with straw removal, straw return significantly increased the rice biomass yield, grain yield, and harvest index by 4.64%, 5.75%, and 4.25% (averaged over two years), respectively. However, the rice yield was lower in the straw return treatment than in the straw removal treatment when the nitrogen fertilizer application rate was ≤90 kg N ha^−1^; the opposite was true when the nitrogen fertilizer application rate was more than 90 kg N ha^−1^. The maximum theoretical grain yield of rice (i.e., the peak of the curve) was calculated by the regression equation, and the optimal nitrogen fertilizer application was found to be 245 kg N ha^−1^ (2020) and 253 kg N ha^−1^ (2021) for the straw removal treatments; the corresponding values for the straw return treatments were 281 kg N ha^−1^ (2020) and 265.8 kg N ha^−1^ (2021). This indicates that continuous straw return can reduce the optimum nitrogen fertilizer application.

### 2.9. Relationships Among Soil Aggregates, Soil Salinity, and Soil Organic Matter

**Figure 10:**
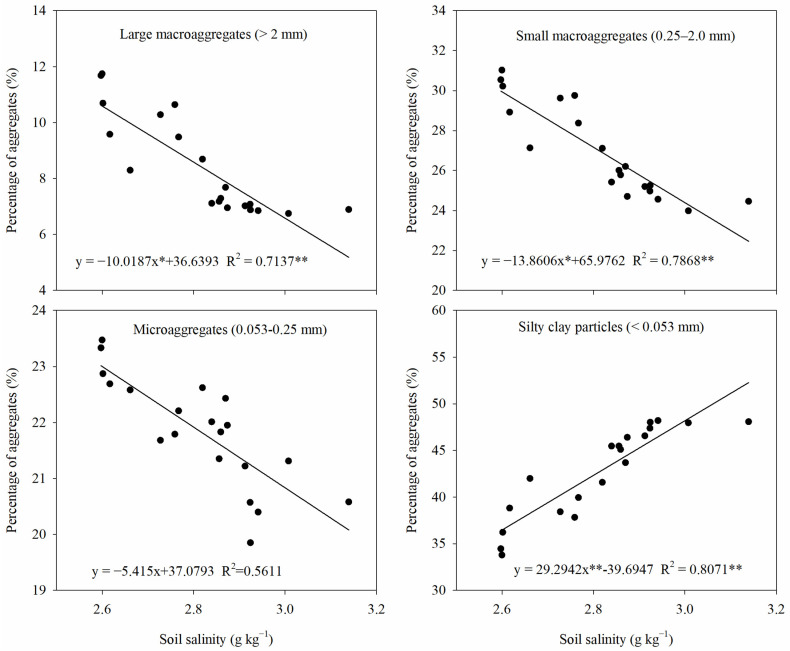
The relationship between soil aggregates of each particle size and soil salinity. * and ** indicate *p* < 0.05 and *p* < 0.01, respectively.

**Figure 11:**
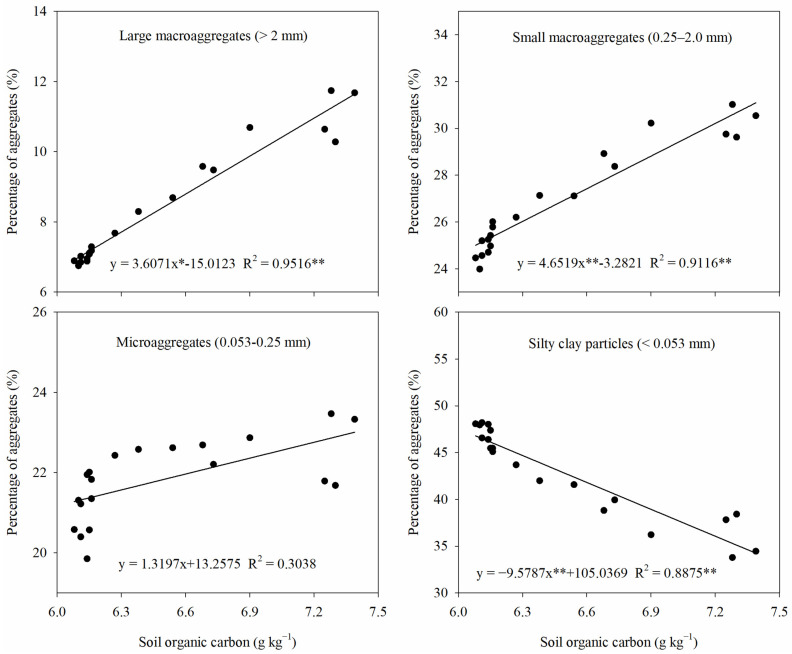
The relationship between soil aggregates of each particle size and soil organic matter. * and ** indicate *p* < 0.05 and *p* < 0.01, respectively.

The relationships among aggregates of different particle sizes, soil salinity, soil organic matter are shown in Figure 10 and Figure 11. Soil salinity was negatively correlated with large macroaggregates, small macroaggregates, and microaggregates and positively correlated with silt clay particles. In particular, there was a significant correlation between soil salinity and large macroaggregates, small macroaggregates, and silty clay particles. In addition, the soil organic carbon content had a significant positive correlation with large macroaggregates and small macroaggregates and a significant negative correlation with silty clay particles. This suggests that changes in soil aggregate structure are closely related to salinity and organic matter in saline–sodic soils.

## 3. Discussion

### 3.1. Combined Effects of Straw and Nitrogen Fertilization on the Physical and Chemical Properties of Soil

Soil salinization is one of the greatest challenges in agricultural production in arid and semi-arid regions and seriously affects local agricultural development. In arid or semi-arid regions, intense evaporation often leads to the accumulation of salts in the upper soil profile; in particular, when there is insufficient leaching, salts move upward rather than downward in the soil profile with water evaporation [24]. Large amounts of Na^+^ in saline soils accumulate in the surface layer, making the soil highly dispersed and leading to the fragmentation of soil aggregates and reduced soil hydraulic conductivity [25]. The specific environment in arid and semi-arid regions makes it impossible to completely eliminate salts from saline soils. Therefore, the focus in improving saline–sodic soils is on improving the structure of the surface soil, diluting its salt content, and enhancing its fertility, to alleviate the degree of salinization of the soil. The use of organic materials has two main beneficial effects in the amelioration of saline soils: the improvement of soil structure and water permeability, which enhances salt leaching, reduces surface evaporation, and inhibits the accumulation of salts in the topsoil [24]. In the present study, straw return significantly reduced the percentage of silty clay particles (<0.053 mm) and also significantly increased the percentage of macroaggregates (>0.25 mm) (Figure 2). This suggests that straw return can promote the formation of large aggregates in saline–sodic paddy soils. This may be for the following reasons: (1) some microaggregates or minute particles attach to the straw after straw return, thus forming macroaggregates [16]; (2) straw return can significantly increase the humus content of saline soil, and the increase in humus content can effectively promote the formation of macroaggregates [16,26], while the organic particles produced by straw degradation can be combined with minerals, which can combine microaggregates and silty clay particles to form macroaggregates [27]; and (3) straw return can significantly increase the activity of fungi and bacteria in the soil, and the secretions that they produce can be used as binders to combine microaggregates and silty clay particles to form macroaggregates [28]. In addition, the interaction of nitrogen fertilizer and straw return was also found to have a significant effect on macroaggregates and silty clay particles in this study (Figure 2). This may be due to the fact that the application of nitrogen fertilizer after straw return can effectively promote the degradation of straw and increase the content of humus and organic matter in saline soils, which in turn promotes the transformation of microaggregates to macroaggregates [14].

**Figure 12:**
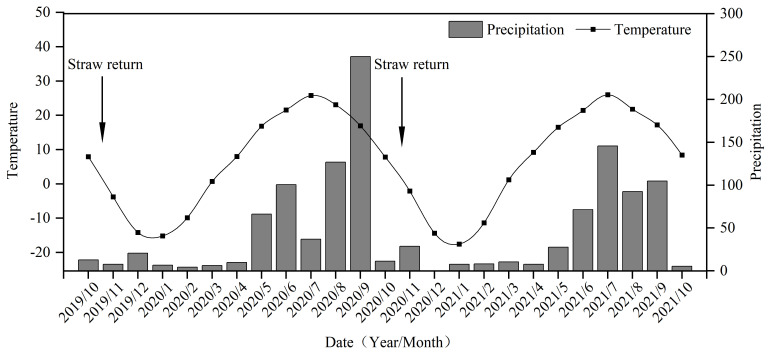
Mean monthly precipitation and temperature during the experiment.

An increase in the proportion of macroaggregates and a decrease in the proportion of silty clay particles in soil will inevitably cause changes in soil bulk density and porosity. Our study also found that straw return significantly reduced the bulk density and significantly increased the soil total porosity in saline–sodic paddy soils, but the application of nitrogen fertilizer had no significant effect on soil bulk density or porosity (Figure 1 and Figure S1). This may be caused by the low temperature (Figure 12) and harsh physicochemical properties of the soil (Table 1) in the experimental area, which led to the slow degradation of straw, while the large amount of straw applied to the soil occupied a large amount of space, dominated by large pores, which reduced the bulk density. Although the application of nitrogen fertilizer promotes straw degradation and reduces the amount of straw per unit volume of soil [14], the degradation of straw increases the soil organic matter content [29], which leads to there being no effect on the soil bulk density or porosity with nitrogen fertilizer application under the same straw return conditions.

Changes in soil structure often cause alterations in soil chemistry. The soil salinity and EC directly reflect surface soil salinity conditions. In the present study, it was found that straw return significantly reduced the salinity and EC_e_ of the topsoil, while the application of nitrogen fertilizer had no significant effect on the salt content of the surface soil (Figure 4). Zhao et al. [30] found similar results in saline dry fields, where they observed a significant decrease in the electrical conductivity of the topsoil after tilling the straw into the 25–30 cm soil layer. This suggests that straw return has a positive effect in reducing salinity in saline–sodic paddy soils. The significant reduction in topsoil salinity after straw return may be attributed to the following factors: (1) Straw return changed the physical structure of the soil in saline–sodic paddy fields (Figure 1 and Figure 2), improved the soil’s water permeability, and effectively increased the salt leaching efficiency, which in turn reduced the salt content of the topsoil [31]. Soil salinity was significantly negatively correlated with macroaggregates (>0.25 mm) and significantly positively correlated with silty clay particles (<0.053 mm) (Figure 10). This again indicates that the improvement of soil structure can reduce soil salinity. (2) Straw return interrupted the continuity of capillary movement of salts from the deeper soil layers and reduced the accumulation of salts in the surface soil [30]. (3) Straw return could significantly increase the organic matter content of saline soil [32], and the increase in organic matter content could significantly inhibit the accumulation of salts in the surface soil [33]. Leogrande and Vitti [34] also showed a definite link between soil salinity, structure, and SOM content. Therefore, we suggest that straw return can reduce salt accumulation in topsoil through different mechanisms in saline–sodic rice fields.

In addition, this study found that straw return significantly reduced the ENa^+^ content in saline–sodic soils (Figure 5). This may be due to the large number of organic acids produced by the straw during degradation [27,35], which promotes the dissolution of calcium carbonate in the soil, thus increasing the amount of Ca^2+^ in the soil solution [36]. This phenomenon leads to a large increase in Ca^2+^ and the replacement of Na^+^ in the soil colloid, and the displaced Na^+^ is discharged with water to the river or to deeper layers of the soil, leading to a decrease in ENa^+^ in the surface soil. Soil pH is closely related to the exchangeable Na^+^ in soil colloids and soil salinity [37]. As straw return significantly reduces the surface soil ENa^+^ and salinity (Figure 4 and Figure 5), it leads to a decrease in soil pH. At the same time, the organic acids released during straw degradation neutralize HCO_3_^−^ and CO_3_^2−^ in the soil [27,35], which also leads to a decrease in soil pH. This study also found that straw return increases the CEC of saline–sodic paddy soils (Figure 5). This may be due to the fact that straw return can significantly increase the humus content in saline soils, and soil humus can adsorb exchangeable cations in the soil solution, such as K^+^, Ca^2+^, and Mg^2+^, which in turn significantly increases the CEC of saline–sodic soils [38].

### 3.2. Combined Effects of Straw and Nitrogen Fertilization on Soil Nutrients

Maintaining soil fertility is essential for sustainable land use. Soil organic matter is commonly used to broadly assess soil fertility because of its ability to directly and indirectly affect plant growth [39]. This study showed that straw return significantly increased soil SOM, TN, AP, and AK contents (Figure 6). This is similar to the findings of Che et al. [40] in saline soils. This indicates that straw return can increase the nutrient content and soil fertility in saline–sodic soils. This may be due to 1) the rapid degradation of straw during the rice growing season, when a large amount of nutrients (C, N, P, K, etc.) in the straw are released directly into the soil, which in turn increases the soil nutrient content [14] and 2) the straw return reducing the concentration of NO_3_^−^, PO_3_^4−^, and K^+^ in the soil leachate, retaining more nutrients in the soil, and resulting in an increase in the content of available nutrients in the soil [41,42]. Yang et al. [43] reached similar conclusions for coastal saline soils. In this study, it was also found that the interaction of straw with nitrogen fertilizer (N × S) significantly influenced the soil SOM, TN, AP, and AK contents, but there was no significant difference between the 270 kg N ha^−1^ and 360 kg N ha^−1^ treatments under straw return (Figure 6). This suggests that the combination of straw and N fertilizer application can improve soil fertility, but there may be a threshold for N fertilizer input. The application of nitrogen fertilizer after straw return can adjust the soil C/N balance, enhance the activity and diversity of soil carbon-sequestering microorganisms, accelerate the degradation of rice straw, and promote the formation of organic matter in the soil [14,44]. However, excessive nitrogen fertilizer application can cause C/N imbalance, inhibit straw degradation, and slow down the rate of soil organic matter increase [14,45], which in turn leads to there being no significant difference between the 270 kg N ha^−1^ and 360 kg N ha^−1^ treatments. The consistent trend in soil nutrients after straw return once again illustrates the importance of adding straw to the soil to maintain SOM and land productivity.

By studying the organic carbon content of aggregates with different particle sizes, the storage pattern of soil organic carbon can be further understood [46]. In this study, it was found that the highest organic carbon content was found in small macroaggregates (0.25–2 mm) (Figure 7). This is mainly due to the fact that the small macroaggregates have a higher specific surface area and they can adsorb organic matter through stronger ligand exchange, as well as cation bridges, meaning that the organic carbon content of this particle-size aggregate is higher than that of other particle sizes [47]. In this study, it was also found that straw return significantly increased the organic carbon content of all particle-size aggregates, and the increase in organic carbon content was higher in macroaggregates (>0.25 mm) than in microaggregates (0.053–0.25 mm) and silty clay particles (<0.053 mm) (Figure 7). Gao et al. [48] also showed that straw return contributed more to the increase in organic carbon content in macroaggregates. This may be caused by the following factors: (1) saturation of the binding carbon pool of silty clay particles after straw return and an increase in added carbon, mainly in large aggregates, leading to a smaller increase in the organic carbon content of silty clay particles [49]; (2) soil organic carbon products increasing after straw return and organic carbon acting as a binder binding silty clay particles and retaining them in aggregates, which meant that the silty clay fraction that was not retained in aggregates only bound particles containing less carbon, and this led to a lesser increase in the organic carbon content of silty clay particles [32].

In addition to analyzing the organic carbon content of aggregates at each particle size, considering the different proportions of aggregates at different particle sizes, it is more direct and scientific to evaluate the contribution of aggregates at each particle size to the total organic carbon of the soil, using carbon stock as an indicator. In this experiment, it was found that straw return significantly increased the organic carbon contribution of soil macroaggregates (>0.25 mm) and significantly decreased the organic carbon contribution of silty clay particles (<0.053 mm) compared to straw removal (Figure 8). This was due to the fact that straw return significantly increased the percentage of macroaggregates (Figure 2) and the organic carbon content of aggregates of each particle size (Figure 7) and also significantly reduced the percentage of silty clay particles (Figure 2), which in turn altered the organic carbon contribution of soil aggregates of each particle size. In addition, soil organic matter showed a significant positive correlation with soil macroaggregates and a significantly negative correlation with soil silty clay particles (Figure 11), which explains this well. In the present study, it was also found that the combination of straw return and nitrogen fertilizer had a significant effect on the contribution of organic carbon to both soil macroaggregates and silty clay particles (Figure 8). The newly added carbon in the soil is more likely to be stored in macroaggregates and thus physically protected, whereas the protection of microaggregates is relatively weak [50]. This leads to an increased contribution of organic carbon from macroaggregates under straw return conditions after the application of nitrogen fertilizer. Although the combined application of straw return and nitrogen fertilizer significantly increased the organic carbon content of the silty clay particles (Figure 7), it also significantly decreased the percentage of silty clay particles (Figure 2), which in turn led to a decrease in their organic carbon contribution. In addition, the nitrogen sequestered in macroaggregates is more inclined to exogenous organic matter, while chemical nitrogen fertilizers are more likely to be sequestered on the surface of powdered clay particles through adsorption [51]. The adsorption sites on the surface of powdered clay particles are limited, and with the increase in chemical nitrogen fertilizer application, it will be easy for the excess chemical nitrogen fertilizer to undergo surface migration and downward leaching migration with water when it reaches saturation. This further illustrates the importance of the rational application of nitrogen fertilizer after straw return.

### 3.3. Combined Effects of Straw and Nitrogen Fertilizer on Rice Yield

Rice was not only subjected to high pH stress, high osmotic stress, and Na^+^ toxicity but also had a very low soil nutrient content in saline–sodic rice fields, which was detrimental to rice growth and resulted in lower yields [8]. In this study, straw return significantly increased the biomass yield, grain yield, and harvest index of rice (Figure 9). This may be due to the fact that straw return improved soil physicochemical properties (Figure 1, Figure 2, Figure 3, Figure 4 and Figure 5), increased soil nutrient content (Figure 6), reduced injury from salinity sodic stress to rice, optimized root physiological properties, promoted root nutrient uptake, and improved nutrient use efficiency, thereby increasing the rice biomass yield, grain yield, and harvest index [8,22]. The present study also revealed that the biomass yield of rice gradually increased with increasing N fertilizer application under the same straw regime, but the highest grain yield of rice was achieved at the 270 kg N ha^−1^ fertilizer application rate (Figure 9). The excessive application of nitrogen fertilizer prolongs the nutrient growth stage in rice, slows down the transfer of nutrients from sources to grains, and reduces the nitrogen fertilizer utilization efficiency, which ultimately leads to this phenomenon [22,52].

Another interesting phenomenon found in this study was that rice yields were lower in the straw return treatment than in the straw removal treatment when the N application rate was ≤90 kg N ha^−1^, while the opposite was true when N application was >90 kg N ha^−1^ (Figure 9). The incorporation of high C/N straw into soil promotes microbial uptake and the utilization of the available nitrogen in the soil, whereas the nutrient content of saline–sodic rice soils is extremely poor (Table 1) and unable to satisfy the nitrogen demands of soil microorganisms under straw return [53]. Low nitrogen application rates cannot effectively mitigate the “nitrogen competition phenomenon” that leads to reduced rice yields, whereas appropriate nitrogen application rates provide more nitrogen while meeting the nitrogen needs of soil microorganisms and rice growth. Moreover, the rapid degradation stage of straw coincides with the tillering stage of rice in saline–sodic rice fields [14]. When the nitrogen application rate is too low, rice tillering is severely inhibited, leading to a reduction in the effective number of rice panicles, which ultimately affects the rice yield [15]. In addition, our study found that rice yield decreased when the nitrogen application rate exceeded 270 kg N ha^−1^ with or without straw return (Figure 9). This suggests that straw return in saline–sodic rice fields needs to be accompanied by an appropriate level of nitrogen fertilizer to maximize rice yield and efficiency. Regression equations showed that the highest theoretical rice yields in the straw removal treatments corresponded to N fertilizer applications of 245 (2020) and 253 (2021) kg ha^−1^, respectively, whereas the highest rice yields in the straw retention treatments were obtained with corresponding N fertilizer values of 281 (2020) and 265.8 (2021) kg ha^−1^, respectively (Figure 9). This indicated that the optimum nitrogen application rate for conventional rice cultivation management was relatively stable, whereas the optimum nitrogen application rate under continuous straw return conditions varied with the number of years of straw return. Since the experiment was conducted for only two years in the field, the positive effects of long-term straw return on soil and rice yield may be greater. Therefore, it is necessary to conduct a long-term study on the combined application of nitrogen fertilizers and straw return to determine the optimal amount of nitrogen fertilizers to be applied at different straw return stages and the actual effect of straw return in saline–sodic rice fields.

## 4. Materials and Methods

### 4.1. Experimental Site

This study was conducted at the Saline–Sodic Experimental Station of Jilin Agricultural University (N 45°35′58″–N 45°36′28″, E 123°50′27″–123°51′31″, 133.7 m a.s.l.), Sheli Town, Baicheng City, Jilin Province, China. This experimental station is located in the western part of Jilin Province, which is a typical representative area of moderately and heavily salinized soil with a temperate continental monsoon climate, and the average annual precipitation, evaporation, hours of sunshine, cumulative temperature, and frost-free period are 413.7 mm (mainly concentrated in June–August), 1696.9 mm, 2996.2 h, 4.7 °C, and 144 days, respectively. The changes in monthly average precipitation and temperature during the experimental period are shown in Figure 12. According to the soil classification system of the World Soil Resource Reference Base [54], the main soils in the experiment are of the Solonetz type. This experiment was conducted over two years (October 2019 to October 2021), and 0–20 cm of soil was collected half a month after the rice harvest in October 2019 to determine the basic physicochemical properties of the soil (Table 1). The experimental plots, which had been deserted soda saline meadow soils before 2015, were reclaimed as rice paddies in 2015. Rice was planted for five consecutive years between 2015 and 2019, and all the straw produced from the rice paddies was burned on-site before spring plowing the following year.

### 4.2. Field Experiment Design

The field experiment was conducted with a two-factor split-plot design with three replications. The main plots were two straw management treatments, straw removal (S0) and straw retention (S), and the subplots were completely randomized to five different N fertilizer applications (0, 90, 180, 270, and 360 kg N ha^−1^). The nitrogen fertilizer application rates in the subplots were set with reference to the study by Yao et al. [55]. Each subplot area was 6 m long and 5 m wide, with an area of 30 m^2^. Each plot was separated by a ridge (0.6 m wide and 0.4 m high) in order to avoid nutrient or water exchange between plots. In addition, each plot had a separate irrigation and drainage system. Nitrogen fertilizer was applied in the ratio of base fertilizer–tillering fertilizer–panicle fertilizer = 6:3:1. Phosphorus fertilizer (P_2_O_5_) and zinc fertilizer (ZnSO_4_) were applied at the same time as the base fertilizer, and the application rates were 50 kg ha^−1^ and 20 kg ha^−1^, respectively. Potassium fertilizer (K_2_O) was applied at a rate of 75 kg ha^−1^ in the ratio of base fertilizer/spike fertilizer = 6:4. N, P, K, and Zn fertilizers were used in the form of urea, Ca(H_2_PO_4_)_2_, KCl, and ZnSO_4_·7H_2_O, respectively. Seeds were sown on 13 April and transplanted on 23 May in 2020 and were sown on 5 April and transplanted on 17 May in 2021. During the experimental period, rice seedlings were transplanted at a density of 30 cm × 16.5 cm, with 3–4 seedlings per hole. The paddy fields were kept flooded at a depth of 3–5 cm from the time of transplanting until 2 weeks before the rice harvest. Diseases, pests, and weeds were strictly controlled during the experimental period to prevent the loss of rice biomass and yield.

After the rice harvest, straw was collected from all test plots by hand. Rice straw was air-dried under natural conditions and then cut into 5–7 cm pieces by a small straw chopper. To avoid the effect of tillage on the experiment, the straw removal plots and straw retention plots were plowed with a reverse stubble rotavator (1GFM-220, Lianyungang Xingan Machinery Manufacturing Co., Ltd., Lianyungang, China) about 20 days after the rice harvest. In straw removal plots, rice was harvested, and all straw was removed from the field with a harrow and plowed with a reverse stubble rotavator. In plots where rice straw was retained, the crushed rice straw was spread evenly over the harvested field surface, and then the rice straw was mixed into the soil at a depth of 0–20 cm using a reverse stubble rotavator. The amount of straw returned was converted according to the local rice yield and the grain-to-straw ratio of 1:1.1, giving 8 t ha^−1^ [56]. For better transplantation, all plots were plowed and slurried again in early May of the following year, and base fertilizer was applied at the time of slurrying.

### 4.3. Sampling and Measurements

Each year after rice harvest, soil samples were collected from 0 to 20 cm using the five-point sampling method, with a 3 m interval between each sampling point, and mixed into one soil sample of about 300 g for the analysis of the soil aggregate structure, chemical properties, and soil nutrients. Each soil sample was carefully removed from the debris and plant and animal residues and passed through an 8 mm sieve before being air-dried in a naturally ventilated room. About 150 g of the air-dried soil samples was used to analyze the soil aggregate structure and aggregate stability, while the rest of the soil samples were used to analyze the chemical properties and nutrients of the soil.

The soil bulk density (BD) was determined using the ring knife method 15 days after rice harvest. The operation was as follows: A 100 cm^3^ volume ring knife was used to cut unstirred soil samples of 0–20 cm in the natural state so that the soil was tightly packed, then three replicates were taken from each plot and then dried at 105 °C. Weighing was carried out; the difference in weight between two replicates was not more than 0.1 g, and the weight per unit of volume was the soil bulk density [6]. The total porosity (TP) of the soil was calculated from the BD using the following equation:

$$\mathrm{TP}\left(\%\right)=\left(1-\frac{\rho a}{\rho r}\right)\;\times \;100$$

where ρa and ρr are the soil bulk weight and 2.65 g cm^−3^.

The wet sieve method was used to size stable soil aggregates [32]. Briefly, 50 g of dry soil was sieved through a series of sieves (2.0, 0.25, and 0.053 mm). After sieving, four aggregate fractions were obtained: large macroaggregates (>2 mm, LM), small macroaggregates (0.25–2.0 mm, SM), microaggregates (0.053–0.25 mm, MI), and silty clay particles (<0.053 mm, CS). The mean weight diameter (MWD) and geometric mean diameter (GMD) were selected as the evaluation indices for aggregate stability [57]. Their calculation formulas were as follows:

$$\mathrm{MWD}=\sum_{i=1}^{n}\bar{X_{i}}W_{i}$$

$$\mathrm{GMD}=\exp\left[\sum_{i=1}^{n}W_{i}\ln\bar{X_{i}}\right]$$

where n is the number of the particle size level of soil aggregates (>2.0 mm, 2–0.25 mm, 0.25–0.053 mm, or <0.053 mm), Di is the mean diameter of the aggregates of each particle size level (2.0 mm, 1.125 mm, 0.152 mm, and 0.053 mm), and W_i_ indicates the proportion of soil aggregate weights in the corresponding size.

The soil pH and soil electrical conductivity (EC_1:5_) were determined by using the potentiometric method with a water–soil ratio of 5:1 as follows: 30 g of air-dried soil was sieved through a 1 mm sieve was weighed and dissolved in 150 mL of distilled water, oscillated, then left to stand for 30 min, and then measured using a pH meter and EC meter [58]. The electrical conductivity of the soil saturation extract (EC_e_) was calculated from the EC_1:5_ with the following formula [59]:

$${\mathrm{EC}}_{e}={EC}_{1:5}\;\times \;10.88$$

where EC_1:5_ is the electrical conductivity of soil with a soil–water ratio of 1:5 in dS m^−1^. EC_e_ is the electrical conductivity of the soil saturation extract in dS m^−1^.

The salinity in air-dried soil samples was determined by using the gravimetric method [60]. Briefly, 50 mL of soil leachate with a soil–water ratio of 1:5 was aspirated and evaporated on a porcelain evaporating dish in a water bath, and the organic matter was completely oxidized via the repeated addition of 150 g L^−1^ of H_2_O_2_, dried at 105 °C, and weighed to obtain the weight of the salts. The soil cation exchange capacity (CEC) was determined using the EDTA–ammonium rapid method [61]; the soil exchangeable sodium (ENa^+^) content was determined via atomic absorption spectrophotometry (NovAA300, Jena Analytical Instruments AG, Jena, Germany) [62]. The soil exchangeable sodium percentage (ESP) was calculated according to the following equation [63]:

$$\mathrm{ESP}\left(\%\right)=\frac{{\mathrm{ENa}}^{+}}{\mathrm{CEC}}\;\times \;100$$

The soil organic matter (SOM) was determined using the digestion method with K_2_Cr_2_O_7_–H_2_SO_4_ [58]. The soil total nitrogen (TN) content was determined using the Kjeldahl method [58]. The soil available phosphorus (AP) content was extracted using 0.5 M NaHCO_3_ and then determined using the molybdenum antimony anti-colorimetric method [58]. The soil available potassium (AK) was extracted with 1 M ammonium acetate and determined using a flame photometer [58].

Rice was harvested from 1 to 3 October each year throughout the experimental period, and three points were randomly selected from each plot, each with a harvest area of 2 × 2 m (except for boundaries and sampling zones), to determine the rice biomass and grain yields per unit area (t ha^−1^). The harvested rice grain yield was adjusted to 14.0% grain moisture content. Meanwhile, the harvest index was calculated based on the biomass yield and grain yield of the rice.

### 4.4. Statistical Analysis

The data obtained in the experiment were organized and basically processed using Microsoft Excel 2016 software. The data for each index between different treatments were analyzed using SPSS 22.0 software, including ANOVA, multiple comparisons, and correlation analysis. The data for each treatment were analyzed to determine the significance of differences using the LSD method at the level of *p* < 0.05. Sigmaplot 14.0 software was used to make figures.

## 5. Conclusions

The soil aggregate distribution, aggregate stability, nutrient status, and rice yield were significantly affected by straw and by straw–nitrogen fertilizer interactions. The increase in rice yield after straw return may be due to the significant improvement in the soil’s physical structure, chemical properties, and nutrient status. In straw return, there was no significant difference in soil properties between 360 kg N ha^−1^ and 270 kg N ha^−1^, but the lowest rice harvest index was recorded at 360 kg N ha^−1^, which resulted in a significantly lower grain yield at 360 kg N ha^−1^ than at 270 kg N ha^−1^. The highest rice grain yield, averaging 8.01 t ha^−1^, was obtained with straw return at 270 kg N ha^−1^ during the experiment. Therefore, straw return combined with 270 kg N ha^−1^ can be considered an effective way to increase rice yield and facilitate soil improvement in saline–sodic rice fields. However, regression analysis showed that the optimal N fertilizer application rate in 2021 (265.8 kg ha^−1^) was lower than that in 2020 (280 kg ha^−1^) under straw return conditions. This suggests that continuous straw return may reduce the optimal nitrogen fertilizer application rate.

## Figures and Tables

**Table 1:** Basic physicochemical properties of the soil in the test plots.

Parameter	Unit	Mean
Sand	%	12.2
Silt	%	65
Clay	%	22.8
Bulk density (BD)	g cm^−3^	1.52
pH	-	8.91
Electrical conductivity of soil saturation extract (EC_e_)	dS m^−1^	12.58
Soil salinity	g kg^−1^	3.29
Exchangeable sodium (ENa^+^)	cmol_c_ kg^−1^	5.34
Cation exchange capacity (CEC)	cmol_c_ kg^−1^	14.11
Exchangeable sodium percentage (ESP)	%	37.87
Total N	g kg^−1^	0.32
Organic matter	g kg^−1^	6.08
Available P	mg kg^−1^	8.84
Available K	mg kg^−1^	102.34

Note: The methods for the determination of soil basic parameters are described in Section 4.3.

## Data Availability

Data will be made available on request.

## Supplementary Materials

The following supporting information can be downloaded at: https://www.mdpi.com/article/10.3390/plants13243475/s1, Figure S1: Effect of straw return with nitrogen fertilizers on bulk density (A and B) and total porosity (C and D) of paddy soils.
