# Supplementary material for: Effects of Straw Management and Nitrogen Application Rate on Soil’s Physicochemical Properties and Rice Yield in Saline–Sodic Paddy Fields
Source: Plants (Basel). 2024 Dec 11;13(24):3475. doi: 10.3390/plants13243475 (PMC11728512; doi:10.3390/plants13243475)
Supplement: Supplementary file 1 [file plants-13-03475-s001.zip › plants-3303856-supplementary.pdf]

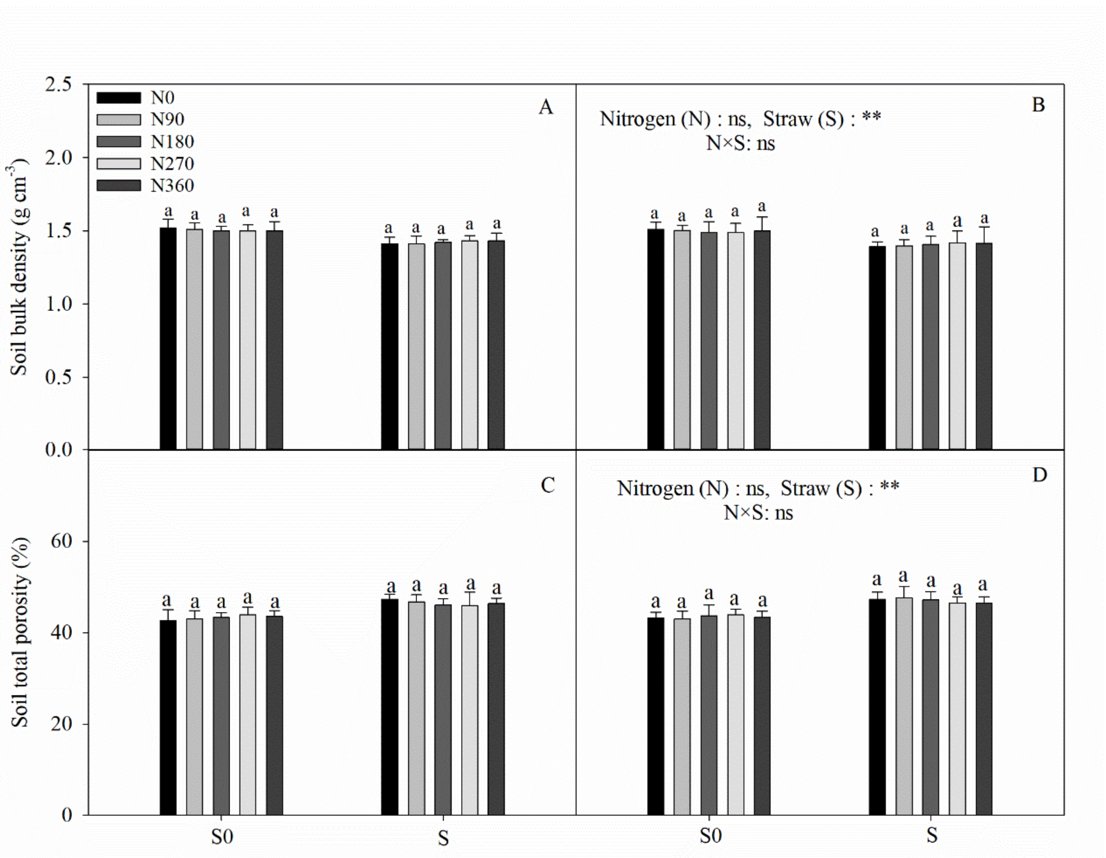

Figure S1. Effect of straw return with nitrogen fertilizers on bulk density (A and B) and total porosity (C and D) of paddy soils. Data in the figures are mean ± standard error. The different lowercase letters in the figure for the same straw management system indicate that the values are significantly different at the 0.05 level. S0, S and N are straw removal, straw retention and nitrogen fertilizer, respectively. N0, N90, N180, N270, and N360 are nitrogen applications of 0, 90, 180, 270, and 360 kg ha^{-1}, respectively. A and C denote 2020, B and D denote 2021. ** mean $p < 0.01$; ns means no significant.
